# Hepatitis B virus reactivation after radiotherapy for hepatocellular carcinoma and efficacy of antiviral treatment: A multicenter study

**DOI:** 10.1371/journal.pone.0201316

**Published:** 2018-07-30

**Authors:** Baek Gyu Jun, Young Don Kim, Sang Gyune Kim, Young Seok Kim, Soung Won Jeong, Jae Young Jang, Sae Hwan Lee, Hong Soo Kim, Seong Hee Kang, Moon Young Kim, Soon Koo Baik, Minjong Lee, Tae-Suk Kim, Dae Hee Choi, Sang-Hyeon Choi, Ki Tae Suk, Dong Joon Kim, Gab Jin Cheon

**Affiliations:** 1 Department of Internal Medicine, University of Ulsan College of Medicine, Gangneung Asan Hospital, Gangneung, South Korea; 2 Department of Internal Medicine, Soonchunhyang University College of Medicine Bucheon Hospital, Bucheon, South Korea; 3 Department of Internal Medicine, Soonchunhyang University College of Medicine Seoul Hospital, Seoul, South Korea; 4 Department of Internal Medicine, Soonchunhyang University College of Medicine Cheonan Hospital, Cheonan, South Korea; 5 Department of Internal Medicine, Yonsei University Wonju College of Medicine, Wonju, South Korea; 6 Department of Internal medicine, Kangwon National University Hospital, Chuncheon, South Korea; 7 Department of Internal Medicine Hallym University College of Medicine, Chuncheon, South Korea; Centre de Recherche en Cancerologie de Lyon, FRANCE

## Abstract

Convincing data that support routine use of preventive therapy against hepatitis B virus (HBV) reactivation in radiotherapy (RT) for hepatocellular carcinoma (HCC) are lacking. The aim of this study was to investigate the incidence, clinical significance, and risk factors of HBV reactivation after RT. Medical records of 133 HBsAg (+) HCC patients who received radiotherapy from March 2009 to February 2016 were reviewed. Patients were divided into two groups: 1) non-antiviral group, those who did not receive antiviral therapy before RT (n = 27); and antiviral group (those who underwent antiviral therapy before RT) (n = 106). Factors related to HBV reactivation in HCC patients were evaluated. 17 (12.7%) of 133 patients developed HBV reactivation after RT. Patients in the antiviral group had significantly lower rates of HBV reactivation than those in the non-antiviral group (7.5% vs. 33.3%, p<0.001). HBV related hepatitis was also lower in the antiviral group (3.8% vs. 14.8%, p = 0.031). In multivariate analysis, absence of antiviral treatment (OR: 8.339, 95% CI: 2.532–27.470, p<0.001) and combined treatment of RT with transarterial chemoembolizatoin (TACE) (OR: 5.313, 95% CI: 1.548–18.232, p = 0.008) were risk factors for HBV reactivation. HBV reactivation can occur after radiotherapy. Combination treatment of RT with TACE and non-antiviral treatment are major risk factors for HBV reactivation during or after RT. Therefore, preventive antiviral therapy should be recommended for patients with HCC who are scheduled to receive RT.

## Introduction

Reactivation of hepatitis B virus (HBV) is a well-known complication in chronic hepatitis B patients receiving immunosuppressive therapy [1]. HBV reactivation can lead to asymptomatic increase of serum transaminase levels and acute hepatic failure [2]. Thus, it is clinically important to prevent HBV reactivation in cancer patients. HBV reactivation in cancer patients receiving cytotoxic chemotherapy can lead to premature termination of chemotherapy or delay in treatment schedules [3].

Recently, it has been reported that HBV reactivation can occur in hepatocellular carcinoma (HCC) treatment such as transarterial chemoemblization (TACE), resection, and radiofrequency ablation (RFA) [4–6]. HBV reactivation after TACE in HCC treatment has been well-studied. It has been reported that HBV reactivation can occur in 4%–40% of patients undergoing TACE treatment [4, 7, 8]. HBV reactivation has also been observed in 15.5%–27.7% of patients after surgical resection of hepatocellular carcinoma [5, 9, 10]. Previous study has suggested that RFA can reactivate HBV replication. However, the incidence of HBV reactivation after RFA is relatively lower (5.6%) than that after hepatic resection (14.0%) [6]. HBV reactivation during HCC treatment can affect liver function and survival [10–12]. To reduce HBV reactivation and reserve liver function after TACE, resection, and RFA, antiviral therapy is very important. Radiotherapy (RT) was infrequently performed for HCC due to radiation induced liver disease (RILD) [13]. There is no effective treatment after the development of RILD. Recently, RT has been used to treat patients with HCC who are ineligible for loco-regional therapy [14, 15]. With advances in radiation technology, RILD after RT treatment is tolerable [16]. However, the risk of HBV reactivation after RT in HCC patients remains unclear.

Thus, the objective of this study was to determine the incidence and risk factors of HBV reactivation after RT in HCC patients. We also evaluated the effect of antiviral therapy on HBV reactivation and clinical outcome correlated with HBV reactivation.

## Materials and methods

### Patients

Between March 2009 and February 2016, HCC patients treated with RT at seven centers were reviewed in this retrospective study. All patients underwent tests for HBsAg, antibody to HBsAg (anti-HBs), hepatitis B e antigen (HBeAg), antibody to HBeAg (anti-HBe), antibody to hepatitis B core antigen (anti-HBc), serum HBV DNA quantification, liver function (alanine aminotransferase [ALT], ALP, total bilirubin, and albumin, prothrombin time) before and after RT. We excluded the following patients: 1) HBsAg negativity; 2) evidence of co-infection with human immunodeficiency virus and hepatitis C virus; 3) loss to follow-up within 3 months after RT; 4) No available HBV DNA quantification before and after RT; 5) history of glucocorticoid and immunotherapy. All patients were followed up at intervals of less than 3 months. Post RT HBV DNA levels were checked during follow-up less than 3 months. Initial HBV DNA levels after RT were checked in 17 patients at 0–1 months, 46 patients at 1–2 months, and 70 patients at 2–3 months.

A total of 464 patients received RT for HCC. Among these, we excluded 179 HBsAg negative patients. Of the remaining 285 patients, 152 patients were excluded from analysis due to no available HBV DNA level, follow-up loss, or poor data integrity within 3 months after the RT. Finally, a total of 133 patients with HBV-HCC were enrolled in the study. This study was approved by the Institutional Review Board of each tertiary center (reference number: 2016-06-13) and written informed consent was waived because of the retrospective study.

### Radiotherapy

RT was considered for patients who had post TACE progression or was not eligible for resection or RFA. RT was performed by two methods: conventional RT (CRT) and stereotactic body RT (SBRT). In CRT, three-dimensional conformal radiotherapy was usually used to treat medium- or large-sized HCC. Each patient received a daily fraction of 1.8–2.0 Gy up to a total dose of 45–64 Gy in five fractions per week up to 25–32 fractions. In SBRT, the total dose administered was 40–60 Gy in three to five fractions over consecutive days or twice a week to treat small-sized HCC.

### Antiviral therapy

Antiviral therapy was carried out according to the Korean Association for the Study of the Liver (KASL) guideline [17]. Antiviral therapy was administered in the HCC patients who had an ALT > upper limit of normal (UNL) (>40 IU/L) and detectable levels of HBV DNA (>20 IU/ml). Patients who had ALT < UNL or undetectable levels of HBV DNA were performed RT without antiviral treatment. Patients were divided into two groups based on antiviral treatment during RT: 1) antiviral group, patients with antiviral treatment during RT; and 2) non-antiviral group, patients without antiviral treatment during RT. Four types of oral antiviral drugs were used: 100mg lamivudine (Heptodin, GlaxoSmithkline), 10mg adefovir dipivoxil (Hepsera GlaxoSmithkline), 0.5mg entecavir (Baraclude, Sino-American Squibb Pharmaceuticals) and 300mg tenofovir (Viread, Gilead Sciences).

### Definition

HBV reactivation was defined as a >10-fold increase in HBV DNA level compared to baseline or the appearance of HBV DNA from an undetectable level at baseline and a post-treatment HBV DNA > 200 IU/mL [4]. Hepatitis was defined as a threefold or more increase in serum ALT level above the upper limit of normal (< 40 IU/L) or an absolute increase of ALT to more than 100 IU/L. RILD was defined as worsening of Child-Pugh (CP) score by 2 or more within 3 months after RT [18]. Hepatitis attributed to HBV reactivation was defined as the presence of hepatitis in patients with HBV reactivation. RILD attributed to HBV reactivation was defined as the presence of RILD in patients with HBV reactivation. TACE combined with RT was defined as performing RT within 4 weeks after TACE [19].

### Statistical analyses

Baseline data were analyzed using Student’s *t*-test, Chi-square test, and Fisher’s exact test when appropriate. Univariate analysis and multivariate analysis using logistic regression model were performed to identify risk factors for HBV reactivation. Data were analyzed using SPSS version 18.0 (SPSS Inc., Chicago, IL, USA).

## Results

### Patient characteristics

A total of 133 HCC patients were treated with RT in this study, including 106 patients who received antiviral treatment during RT (antiviral group) and 27 patients who did not receive antiviral treatment during RT (non-antiviral group). Of the 106 patients who received antiviral treatment, 18 received lamivudine 100 mg per day, 6 received adefovir dipivoxil 10 mg per day, 9 took lamivudine 100 mg with adefovir dipivoxil 10 mg per day, 52 took entecavir 0.5 mg per day, and 21 took tenofovir 300 mg per day. There were no significant differences in baseline characteristics between the antiviral group and the non-antiviral group. Baseline characteristics of these 133 patients are summarized in Table 1.

**Table 1:** Baseline characteristics of patients in the antiviral group and the non-antiviral group.

Variable	Antiviral group (n = 106)	Non-antiviral group (n = 27)	P value
Sex			
Male/Female	83/23	22/5	0.718
Age (Year)	58.8 ± 9.7	57.3 ± 6.6	0.455
ALT	42.5 ±34.5	33.0 ± 19.9	0.171
AFP (median)	32.4 (1–3328000)	45.0 (2.03–4640000)	0.367
HBeAg			
Positive/negative	20/86	3/24	0.341
HBV DNA (IU/ml)			
>2000/ <2000	26/80	8/19	0.354
Child pugh class			
A/B	87/19	26/1	0.065
Radiotherapy type			
CRT/SBRT	67/39	16/11	0.705
Radiation dose (Gy)	48.8 ± 0.7	48.1 ± 1.0	0.716
Combination treatment with TACE			
Yes/No	48/58	10/17	0.440
Tumor size	4.3 ± 3.8	5.2± 4.2	0.295

Values are presented as median (range), or mean ± SD.

ALT, alanine aminotransferase; HBV, Hepatitis B virus; AFP, a-fetoprotein; CRT, conventional radiotherapy; SBRT, Stereotactic body radiotherapy; TACE, transarterial chemoembolization.

### Clinical outcomes between patients with or without antiviral treatment

HBV DNA reactivation occurred in 17 (12.7%) of 133 patients after RT. Mean duration of HBV reactivation after RT was 2.3 ± 0.7 months. Those who received antiviral treatment showed lower incidence of HBV reactivation compared to those who received no antiviral treatment (7.5% [8/106] vs. 33.3% [9/27], *P*<0.001). Incidence rate of hepatitis was 16.0% (17/106) in the antiviral group and 29.6% (8/27) in the non-antiviral group (*p* = 0.107). The incidence rate of hepatitis due to HBV reactivation was lower in the antiviral group than that in the non-antiviral group (3.8% [4/106] vs. 14.8% [4/27], *P* = 0.031). Incidence rate of RILD was 14.2% (15/106) in the antiviral group and 18.5% (5/27) in the non-antiviral group (*P* = 0.571). Incidence rates of RILD due to HBV reactivation were not significantly different between the non-antiviral group and the antiviral group (4.7% [5/106] vs. 7.4% [2/27], *P* = 0.576) (Table 2). The duration of antiviral treatment before RT is 21.4 (±21.8) months in patients with HBV reactivation and 22.6 (±21.6) months in patients without HBV reactivation (*P* = 0.882).

**Table 2:** Clinical outcomes for HBV reactivation.

	Antiviral group (n = 106)	Non-antiviral group (n = 27)	Total (n = 133)	P value
All cases of HBV reactivation	8 (7.5%)	9 (33.3%)	17 (12.7%)	< 0.001
All cases of Hepatitis	17 (16.0%)	8 (29.6%)		0.107
HBV reactivation	4 (3.8%)	4(14.8%)		0.031
Other causes	13 (12.3%)	4 (14.8%)		0.723
All cases of RILD	15 (14.2%)	5 (18.5%)		0.571
HBV reactivation	5 (4.7%)	2 (7.4%)		0.576
Other causes	10 (11.1%)	3 (9.4%)		0.793

HBV, hepatitis B virus; RILD, radiation induced liver disease.

### Predictive factors of HBV DNA reactivation after RT

In univariate analysis, age, gender, Child-pugh class, HBeAg status, pretreatment HBV DNA level, or type of RT did not affect HBV reactivation. Only non-antiviral therapy and combined treatment of RT with TACE correlated significantly with HBV DNA reactivation after RT. Multivariate analysis using logistic regression model revealed that non-antiviral treatment (OR: 8.339, 95% CI: 2.532–27.470, p<0.001) and combined treatment of RT with TACE (OR: 5.313, 95% CI: 1.548–18.232, p = 0.008) were associated with HBV reactivation (Table 3).

**Table 3:** Risk factors related to HBV reactivation during radiotherapy.

	Univariate analysis			Multivariate analysis		
	OR	95% CI	P value	OR	95% CI	P value
Age (year)	1.006	0.951–1.063	0.839			
Gender						
Male/Female	0.848	0.253–2.836	0.789			
ALT (IU/L)	1.004	0.990–1.018	0.600			
Child-pugh class						
A	1.000					
B	0.319	0.040–2.552	0.282			
Antiviral treatment						
Yes	1.000					
No	6.125	2.087–17.980	0.001	8.339	2.532–27.470	<0.001
Combination treatment with TACE						
No	1.000					
Yes	3.652	1.206–11.056	0.022	5.313	1.548–18.232	0.008
HBeAg	1.000					
Positive/ Negative	0.603	0.128–2.840	0.522			
Pretreatment HBV DNA (IU/ml)						
≥2000	1.000					
< 2000	0.322	0.070–1.483	0.146			
Radiotherapy type						
CRT	1.000					
SBRT	0.639	0.229–1.779	0.391			
Radiation dose (Gy)	1.000	1.000–1.000	0.993			
AFP						
< 200	1.000					
≥200	1.825	0.653–5.102	0.252	1.553	0.499–4.833	0.448

OR, odds ratio; CI, confidence interval; ALT, alanine aminotransferase; HBV, Hepatitis B virus; TACE, transarterial chemoembolization; CRT, conventional radiotherapy; SBRT, Stereotactic body radiotherapy; AFP, a-fetoprotein.

### HBV reactivation in subgroups

**Fig 1:**
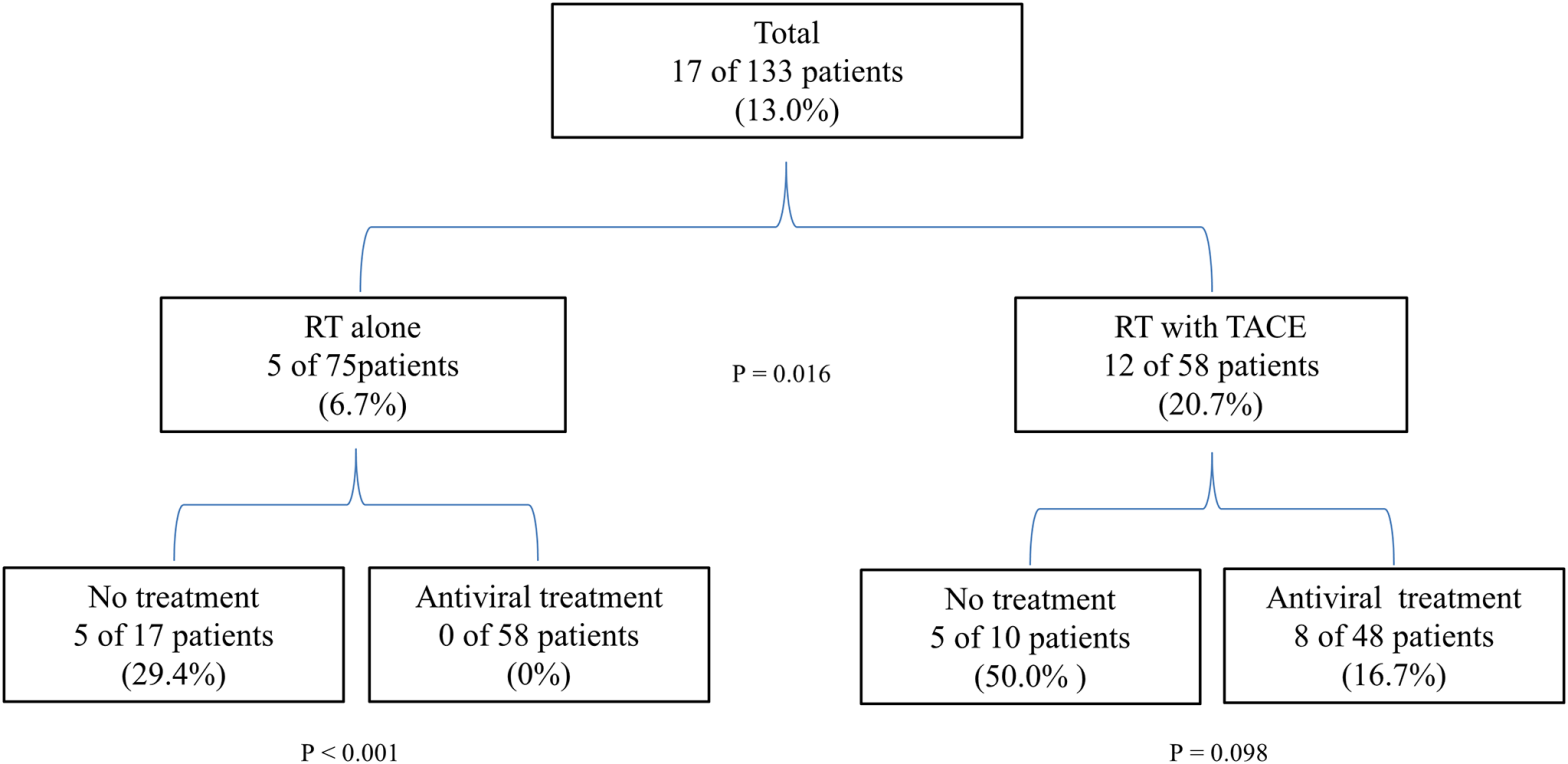
Subgroup analysis according to risk factor related to HBV reactivation during radiotherapy.

Incidences of HBV reactivation were analyzed depending on whether patients received combined TACE treatment before RT. Results are shown in Fig 1. 58 patients received combined treatment of RT with TACE (RT-TACE group) and 75 received RT alone (RT group). There was a significantly higher incidence of HBV reactivation in RT-TACE group than RT group (20.7% [12 of 58] vs. 6.7% [5 of 75], *P* = 0.016).

We also analyzed the effect of antiviral treatment in both RT and RT-TACE groups. In the RT group, the incidence of HBV reactivation was 0% (0 of 58 patients) in those who received antiviral treatment and 29.4% (5 of 17 patients) in those who received no antiviral treatment (*p* < 0.001). In the RT-TACE group, the incidence of HBV reactivation was 16.7% (8 of 48 patients) in those who received antiviral treatment and 50.0% in those who received no antiviral treatment (5 of 10 patients) (*P* = 0.098).

### Clinical features of patients who had HBV reactivation after RT

A total of 21 patients developed HBV DNA reactivation after RT. However, four patients were excluded because the HBV reactivation was due to drug resistance. Clinical features of the remaining 17 patients who developed HBV reactivation after RT are summarized in Table 4.

**Table 4:** Clinical features of 17 patients with HBV reactivation after radiotherapy.

		Treatment				Baseline				Follow up	At the time of reactivation					
	Sex/Age	drug	Combined with TACE	RT Type	RT dose	HBeAg status	ALT	HBV DNA	CP score	Timing of initial HBV DNA after RT (m)	Onset of reactivation after RT (m)	ALT	HBV DNA	CP score	Development of hepatitis	Development of RILD
1	M/47	Lamivudine	Yes	CRT	5040	-	38	< 20	5	2.8	5.6	75	461	5	No	No
2	F/59	Lamivudine	Yes	CRT	5000	-	16	< 20	6	1.2	3.8	155	721	10	Yes	Yes
3	M/54	Lamivudine	Yes	SBRT	4500	-	20	1220	5	2.1	2.1	522	58460	8	Yes	Yes
4	M/64	Adefovir with lamivudine	Yes	CRT	5000	-	23	<20	8	0.8	0.8	241	159	11	Yes	Yes
5	M/69	Adefovir	Yes	SBRT	5200	-	57	<20	5	1.7	1.7	39	415	7	No	Yes
6	M/73	Entecavir	Yes	CRT	4500	+	13	23	5	0.6	3.7	58	1700000	5	Yes	No
7	F/71	Lamivudine	Yes	CRT	4500	-	42	<20	5	2.9	2.9	39	422	6	No	No
8	F/61	Entecavir	Yes	CRT	4500	-	26	<20	5	1.6	1.6	32	144	8	No	Yes
9	M/50	No	Yes	CRT	5000	-	14	1781	5	1.1	3.1	158	1000000	5	Yes	No
10	M/44	No	Yes	SBRT	5400	-	60	3970	5	1.2	1.2	32	135900	9	No	Yes
11	M/50	No	No	SBRT	4800	-	17	1080	5	1.7	1.7	35	89200	5	No	No
12	M/70	No	No	SBRT	6000	-	17	873	5	0.7	0.7	33	155000	6	No	No
13	F/64	No	No	CRT	2700	-	35	<20	5	1.0	1.0	166	2981	10	Yes	Yes
14	M/55	No	No	CRT	5600	-	13	146	6	2.5	2.5	363	52675	5	Yes	No
15	M/54	No	Yes	CRT	4000	-	64	<20	5	1.8	1.8	1316	257	6	Yes	No
16	M/54	No	Yes	SBRT	4500	+	56	25	5	1.4	1.4	67	16200	5	No	No
17	M/63	No	No	SBRT	5040	-	86	20.8	5	1.1	4.2	15	2610	6	No	No

TACE, transarterial chemoembolization; RT, radiotherapy; HBV, Hepatitis B virus; ALT, alanine aminotransferase; CP, child pugh; CRT, conventional radiotherapy; SBRT, Stereotactic body radiotherapy; RILD, radiation induced liver disease.

## Discussion

Our study showed that HBV reactivation could occur after RT. The incidence rate of HBV reactivation was 7.1% in the antiviral group and 31.0% in the non-antiviral group (*P*<0.001, Table 2). We reported that mean duration of HBV reactivation after RT was 2.3 months. This result might be due to the fact that most patients underwent HBV DNA measurement within 2–3 months after RT. Kim et al. first reported HBV reactivation after RT. The cumulative rate of HBV reactivation was 21.8% (7 of 32) in patients who underwent conformal RT without lamivudine therapy [20]. However, their study had limitation in that their sample size was too small. In a study of Huang et al., the incidence rate of HBV reactivation without antiviral treatment was 24.6% at the end of conformal RT [21]. Pathogenesis of RT induced HBV reactivation remains uncertain. According to previous studies, HBV reactivation induced by RT is considered a bystander effect due to cytokines such as IL-6 released by irradiated endothelial cells while IL-6 can induce HBV reactivation through signal transduction pathway [22, 23]. These results suggest that HBV reactivation can occur after RT and antiviral treatment can reduce HBV reactivation after RT.

This study demonstrated that antiviral therapy could reduce the incidence of hepatitis due to HBV reactivation after RT. Hepatitis due to HBV reactivation was significantly lower in the antiviral group compared to that in the non-antiviral group (3.8% [4/106] vs. 14.8% [4/27], *P* = 0.031, Table 2). This suggests that antiviral treatment can prevent hepatitis after RT. It has been reported that preventive antiviral treatment in HBV-HCC patients can reduce side effects after or during HCC treatment and preserve liver function [4, 10, 24, 25]. In a previous study, lamivudine therapy reduced HBV reactivation, hepatitis due to HBV reactivation, and hepatic morbidity during TACE [4]. In a prospective study, Xu et al. have suggested that lamivudine therapy could reduce HBV activation (11.2% vs. 45.6%, *P*<0.001) and improve survival of HCC patients treated with TACE (relative risk = 0.423; 95% CI: 0.248–0.721, *P* = 0.002) [25]. Chen et al. have also reported that patients in the antiviral group have faster (*P* = 0.016) recovery of albumin level [10]. In a randomized control trial, antiviral therapy increased liver function recovery (hazard ratio: 1.23; 95% CI: 0.98–2.55; p = 0.109) [24]. These studies demonstrate that antiviral treatment can reduce treatment-related side effects in patients with HCC. Therefore, antiviral therapy is effective in reducing the risk of HBV reactivation and hepatitis due to HBV reactivation in HCC patients receiving RT.

Our results showed that combined treatment of TACE with RT and non-antiviral treatment were significantly correlated with HBV reactivation in multivariate analysis (Table 3). First, combined treatment of TACE with RT increased the risk of HBV reactivation compared to RT alone (OR: 5.313, 95% CI: 1.548–18.232, p = 0.008), consistent with results of previous studies [26]. In a Korean study, there was a significant trend of increased risk of reactivation with increasing intensity of therapy, with hazard ratio of 1.0 for local ablation therapy, 2.45 for TACE (using Adriamycin), 4.19 for TACE (using cisplatin), and 10.17 for TACE (using cisplatin) with RT [26]. HBV reactivation was also higher in hepatic resection than that in local treatment. Dan et al. have reported that the incidence of HBV reactivation after RFA is relatively lower compared to that after hepatic resection (5.6% vs. 14.0%, p = 0.034) [6]. These results suggest that treatment intensity is correlated with the risk of HBV reactivation. Second, non-antiviral treatment before RT was also associated with high risk of HBV reactivation after RT (OR: 8.339, 95% CI: 2.532–27.470, p<0.001). This result has been well documented in other treatment modalities. Non-antiviral treatment has been reported to be an independent factor for increased risk of HBV reactivation after TACE [4, 9, 25] and after resection [5, 10, 24]. Therefore, non-antiviral therapy and combined treatment of RT with TACE are important risk factor for HBV reactivation in patients receiving RT.

We performed subgroup analysis depending on whether patients received combined TACE treatment before RT. Results are shown in Fig 1. The incidence of HBV reactivation was significantly higher in the combined RT-TACE group compared to that in the RT group (20.7% vs. 6.7%, *P* = 0.016). In the RT group, HBV reactivation did not occur during antiviral treatment. The incidence rate of HBV reactivation in patients without antiviral treatment was 29.4%, similar to results reported in previous studies [20, 21]. Interestingly, the rate of HBV reactivation was 16.7% during antiviral treatment in the TACE-RT group. However, HBV reactivation recovered spontaneously without hepatitis. The rate of HBV reactivation was the highest in patients without antiviral treatment in the TACE-RT group.

This study has some limitations. First, this was a retrospective study. Second, the sample size for the non-antiviral group was small, although we analyzed data from seven institutions. However, we analyzed a large number of patients compared to previous studies. We also analyzed clinical factors to predict HBV reactivation. Third, we did not demonstrate the effect of antiviral therapy on survival in patients with RT. Fourth, we did not show when HBV DNA was reactivated because the timing of HBV measurement after RT was not the same.

In conclusion, HBV reactivation that causes hepatitis can occur after RT in HCC patients. Combined treatment of RT with TACE and non-antiviral treatment are major risk factors for HBV reactivation during RT. Antiviral therapy can reduce HBV reactivation and complications after RT. Therefore, preventive antiviral therapy should be recommended for patients with HCC who are scheduled to receive RT.

## Supporting information

S1 Dataset (XLSX)

S2 Dataset Dataset of reactivation duration. (XLSX)
